# Delayed Post-traumatic Frontal Mucocele Occurrence: A Case Report

**DOI:** 10.7759/cureus.67108

**Published:** 2024-08-18

**Authors:** Alexandros Moniakis, Antonia Malli, Velissarios Smponias, Konstantinos Kasapas

**Affiliations:** 1 Department of Neurosurgery, Athens General Hospital "Georgios Gennimatas", Athens, GRC; 2 Department of Oral and Maxillofacial Surgery, Athens General Hospital "Georgios Gennimatas", Athens, GRC

**Keywords:** delayed, reconstruction, trauma, mucocele, frontal

## Abstract

Mucoceles, benign cystic lesions with pseudo-stratified epithelial lining, typically arise due to chronic sinus ostia obstruction. Mucoceles can cause expansive erosion and, in severe cases, intracranial or intraorbital extension. Here, we present a case of delayed frontal mucocele formation following head trauma, emphasizing the importance of timely diagnosis and surgical intervention. Treatment involves careful mucocele removal and cranialization or obliteration of sinuses. Meticulous reconstruction using various materials ensures optimal cosmetic outcomes.

## Introduction

Mucoceles are mucus-containing benign cystic lesions lined with pseudo-stratified epithelial lining, which usually develop after chronic obstruction of the ostia of paranasal sinuses and may cause expansive erosion of the involved sinus. The sinus that is most commonly involved is the frontal, whereas sphenoid, ethmoid, and maxillary mucoceles are rare [1]. They are regarded as cyst-like expansile and destructive lesions that can spread intracranially as well as intraorbitally [2,3]. In addition, a frontal mucocele can also expand into the subcutaneous tissue and present as a forehead mass [4,5]. The etiology of mucoceles is multifactorial, which involves inflammation, allergy, trauma, anatomic abnormality, previous surgery, fibrous dysplasia, osteoma, or ossifying fibroma [2]. Usually one or more of the above lead to the obstruction of natural ostia which impairs the drainage of the sinus.

Imaging studies along with clinical investigation compose the basis of the diagnostic procedure. Both computed tomography (CT) and magnetic resonance imaging (MRI) provide crucial information for the correct planning of treatment [6]. CT is used in determining the regional anatomy and extent of the lesion, specifically the intracranial extension and the bony erosion, while MRI is useful in differentiating mucoceles from neoplasms via contrast enhancement.

A rare and intriguing cause of mucocele formation is as a complication years after trauma to the frontal sinus [7]. This development is mostly due to compromised ventilation, or in cases of cranialization of the frontal sinus, residual mucosa is the cause of the mucocele formation [7].

These types of lesions are best dealt with by a multidisciplinary team to avoid all possible sequelae [3]. In the case we are reporting, surgery was performed by a team composed of neurosurgeons and maxillofacial surgeons.

## Case presentation

**Figure 1:**
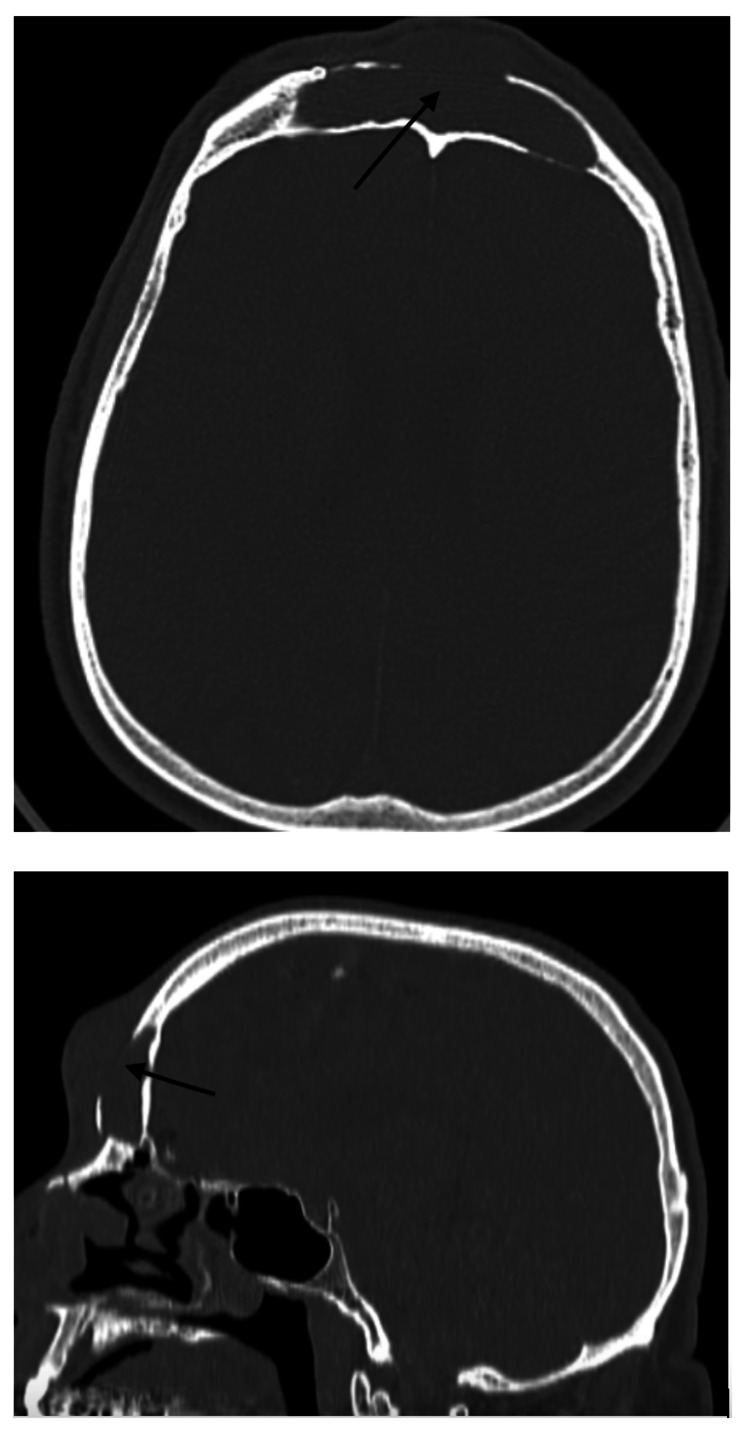
Axial and coronal CT scan showing mass via the destroyed anterior wall of the frontal sinus. CT: computed tomography

**Figure 2:**
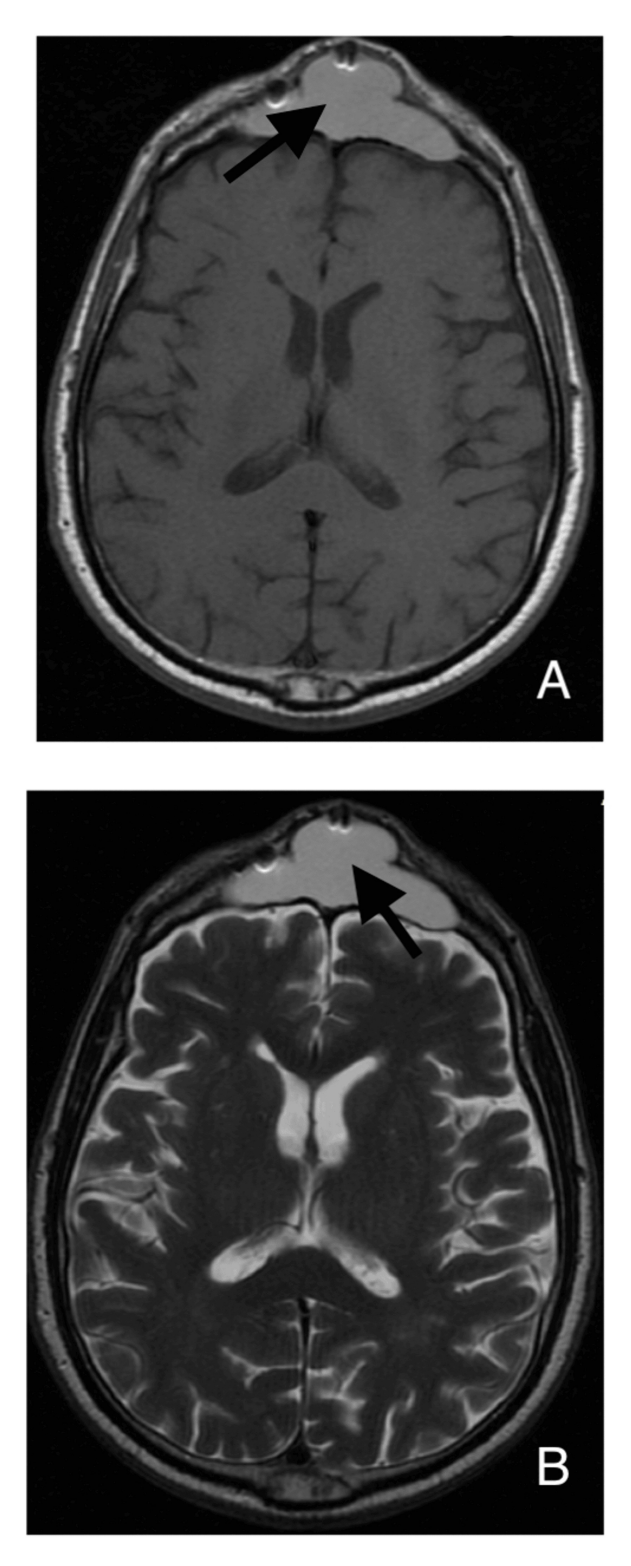
T1 and T2 axial MRI scan showing a hyperdense frontal lesion. MRI: magnetic resonance imaging

A 68-year-old male patient was admitted to our department with a 3-cm frontal swelling with no signs of infection. The patient had a known history of head trauma 20 years ago, and since then, he had observed a small frontal swelling which has increased in size and remained irreducible during the last six months. CT scan initially and MRI subsequently revealed a frontal mass in relation to the frontal sinus and bony erosion of the sinus' anterior wall (Figures 1-2). After completing the work-up, the patient underwent a surgery for exploration.

**Figure 3:**
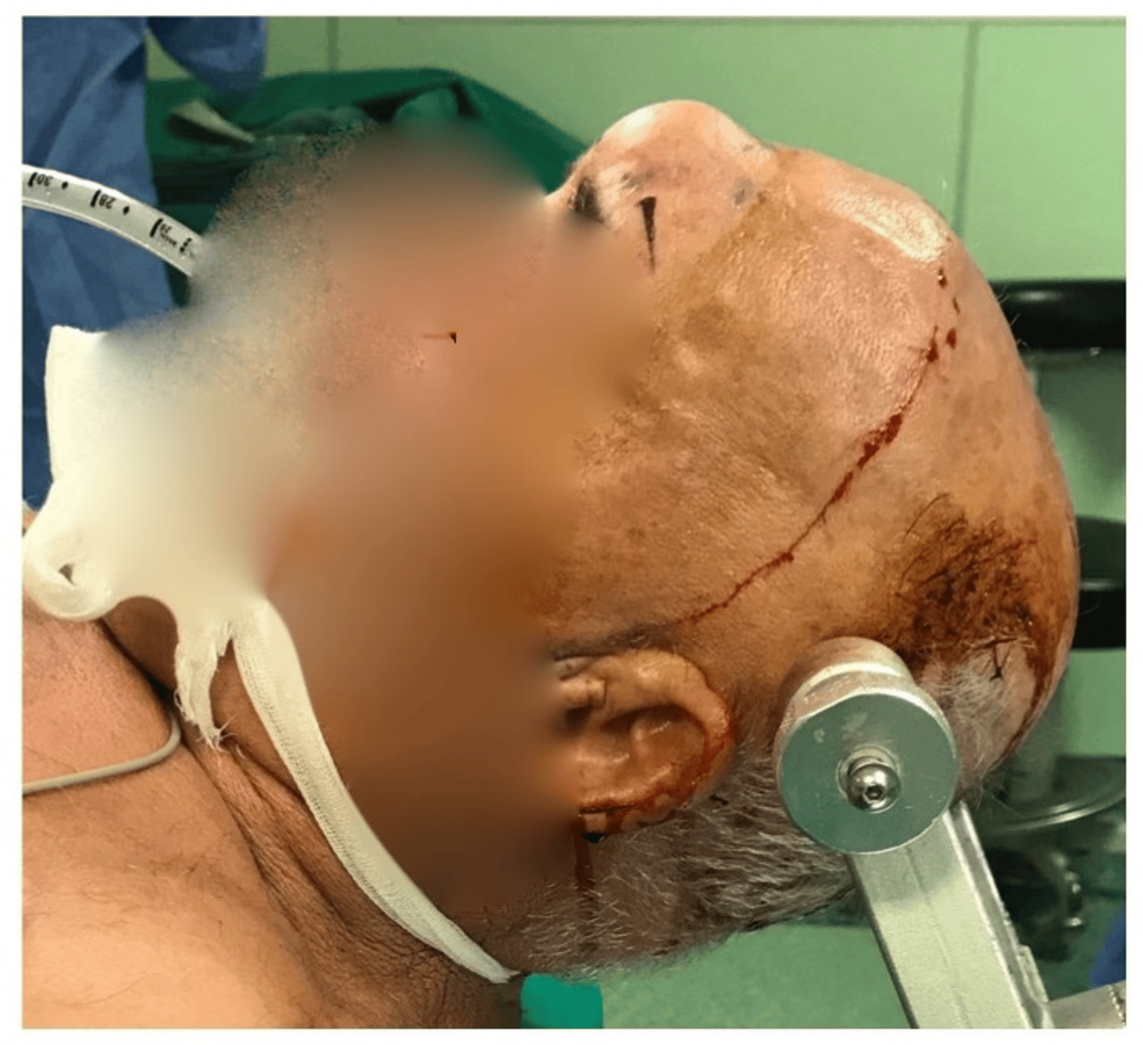
The patient's position with the head placed on a three-pin skull clamp.

**Figure 4:**
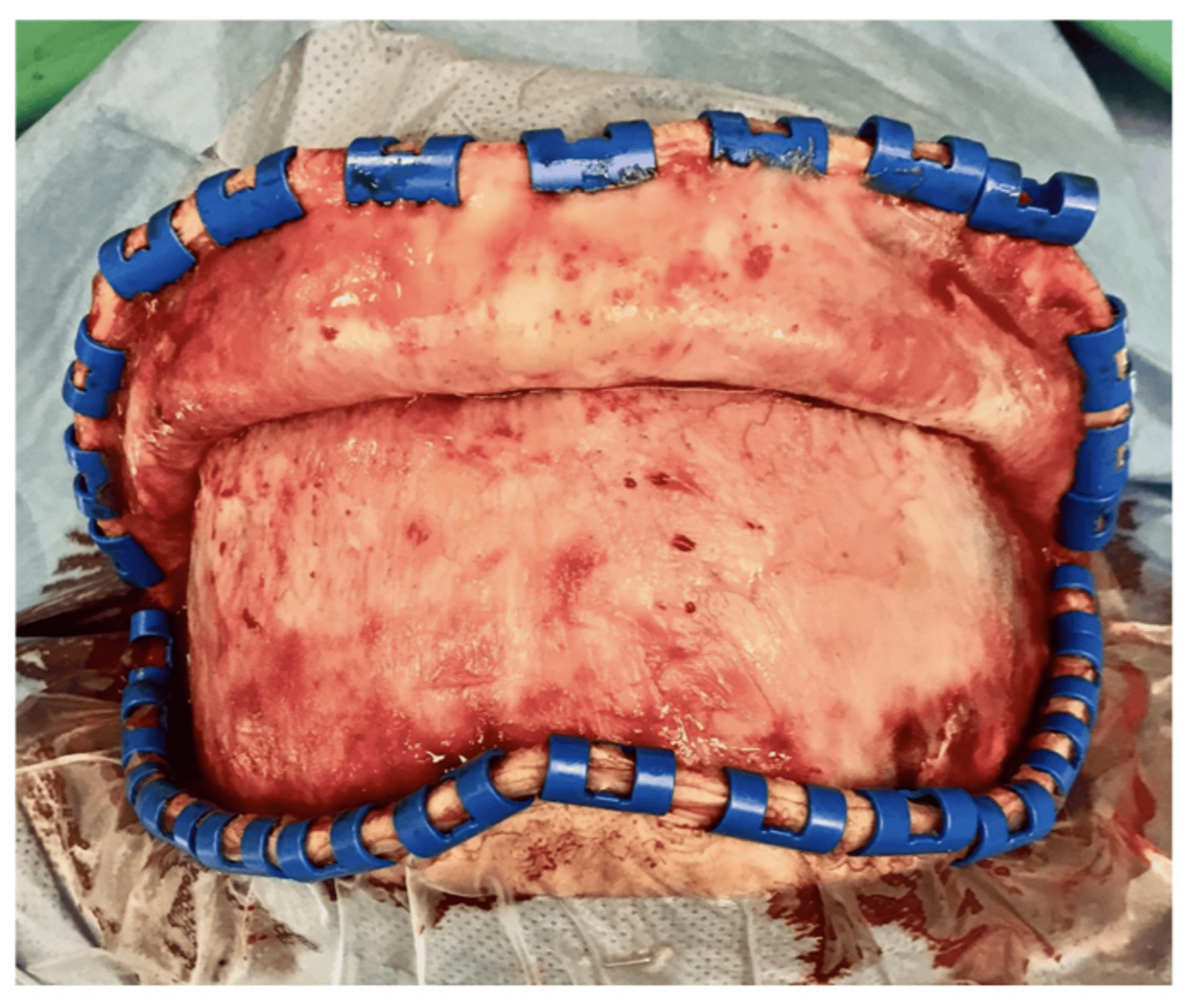
Bicoronal skin incision with pericranium preservation.

**Figure 5:**
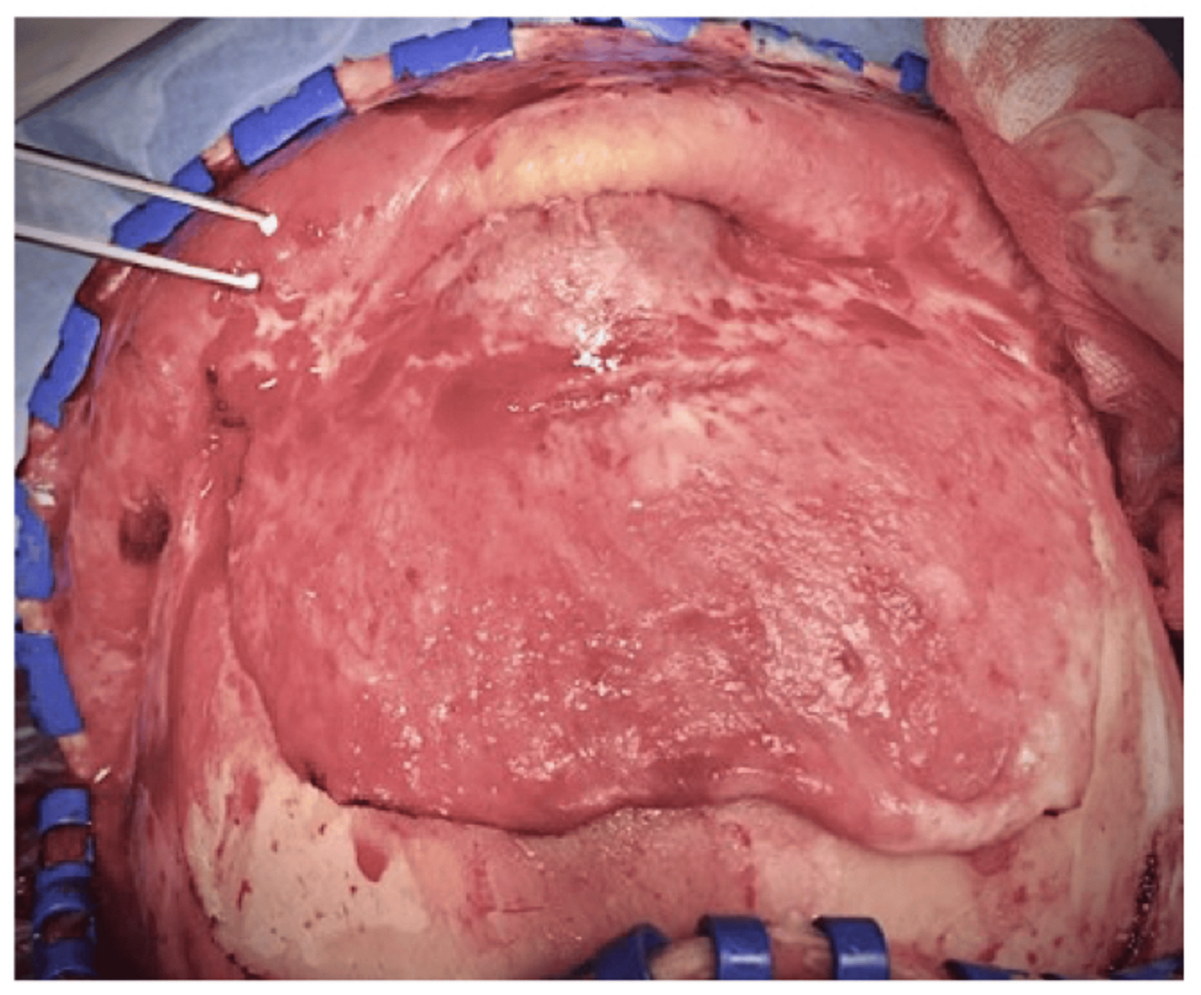
Pericranium flap harvesting and recognition of mass under the flap.

**Figure 6:**
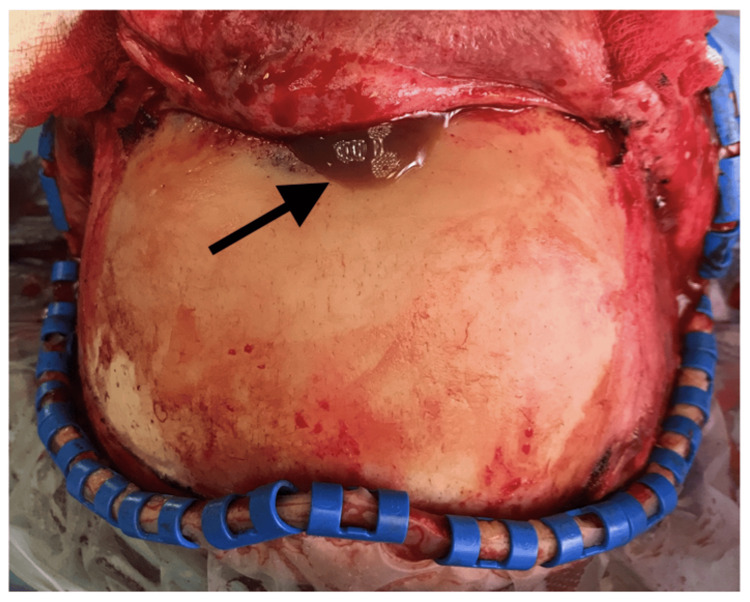
After pericranium flap elevation, the liquified content of the mucocele was recognized.

**Figure 7:**
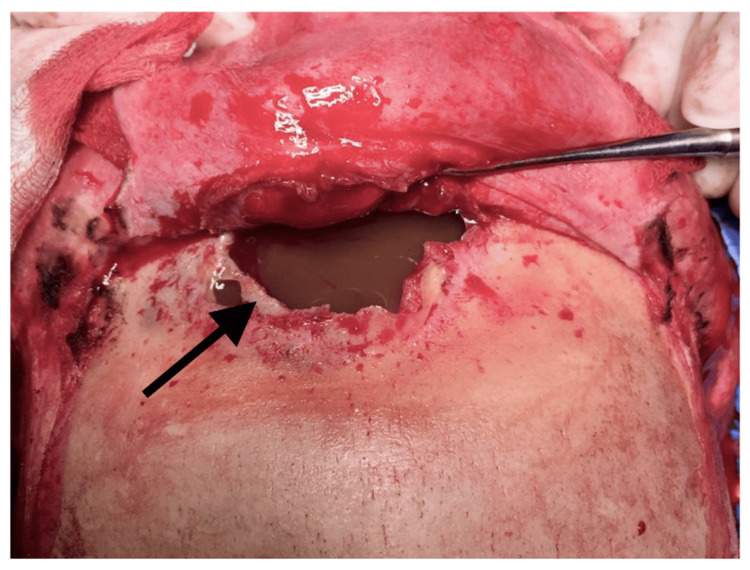
The anterior wall of the frontal sinus was found destroyed.

**Figure 8:**
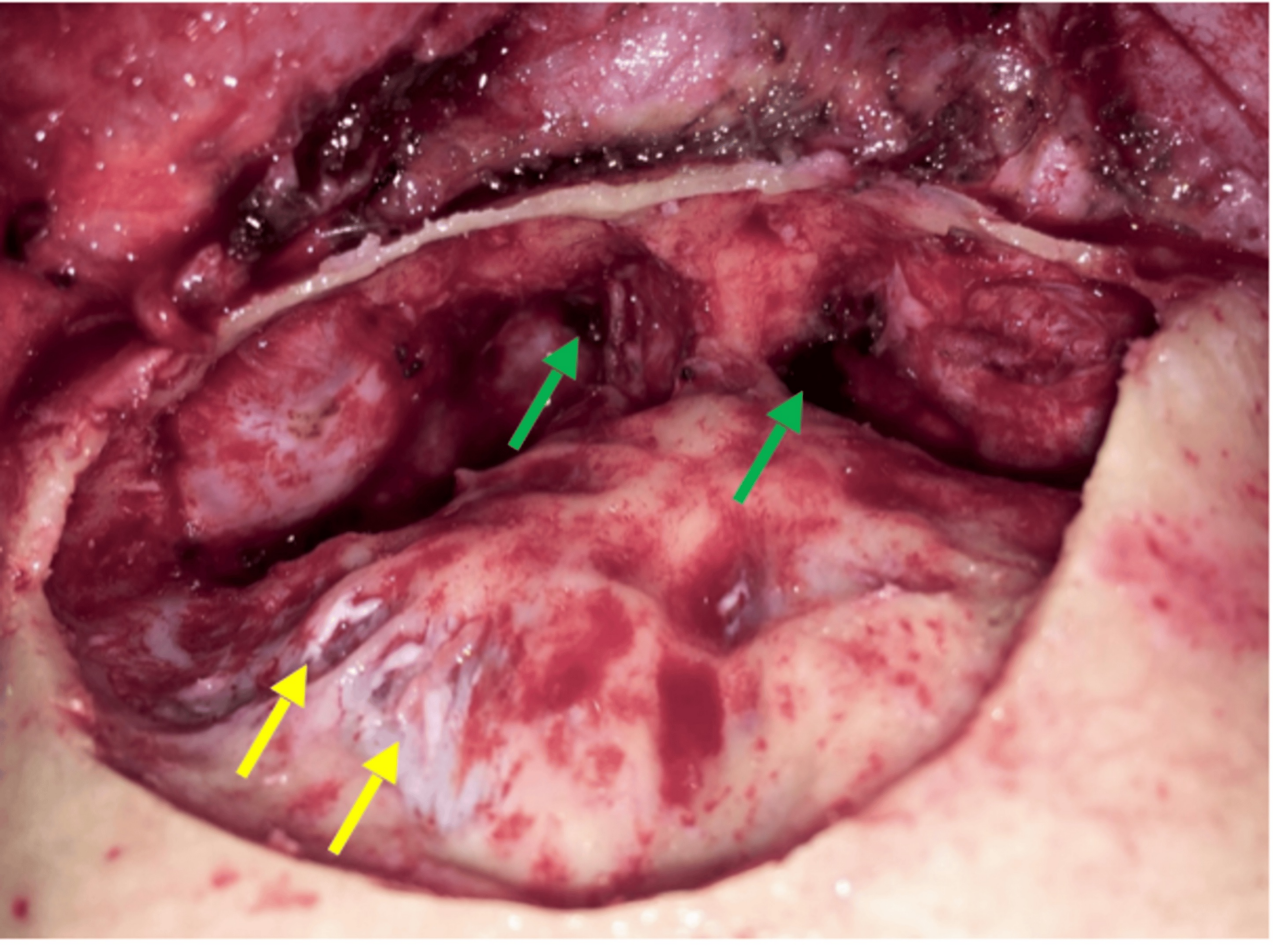
Drilling of the anterior wall revealed the posterior wall of the enlarged frontal sinus. The frontal ostium and recesses were also bilaterally identified (green arrows). The mucosa has been meticulously removed from both the sinus cavity and the frontal recesses to prevent any recurrence. The dura was found to be exposed due to a bone erosion of the left posterior wall of the frontal sinus (yellow arrows).

**Figure 9:**
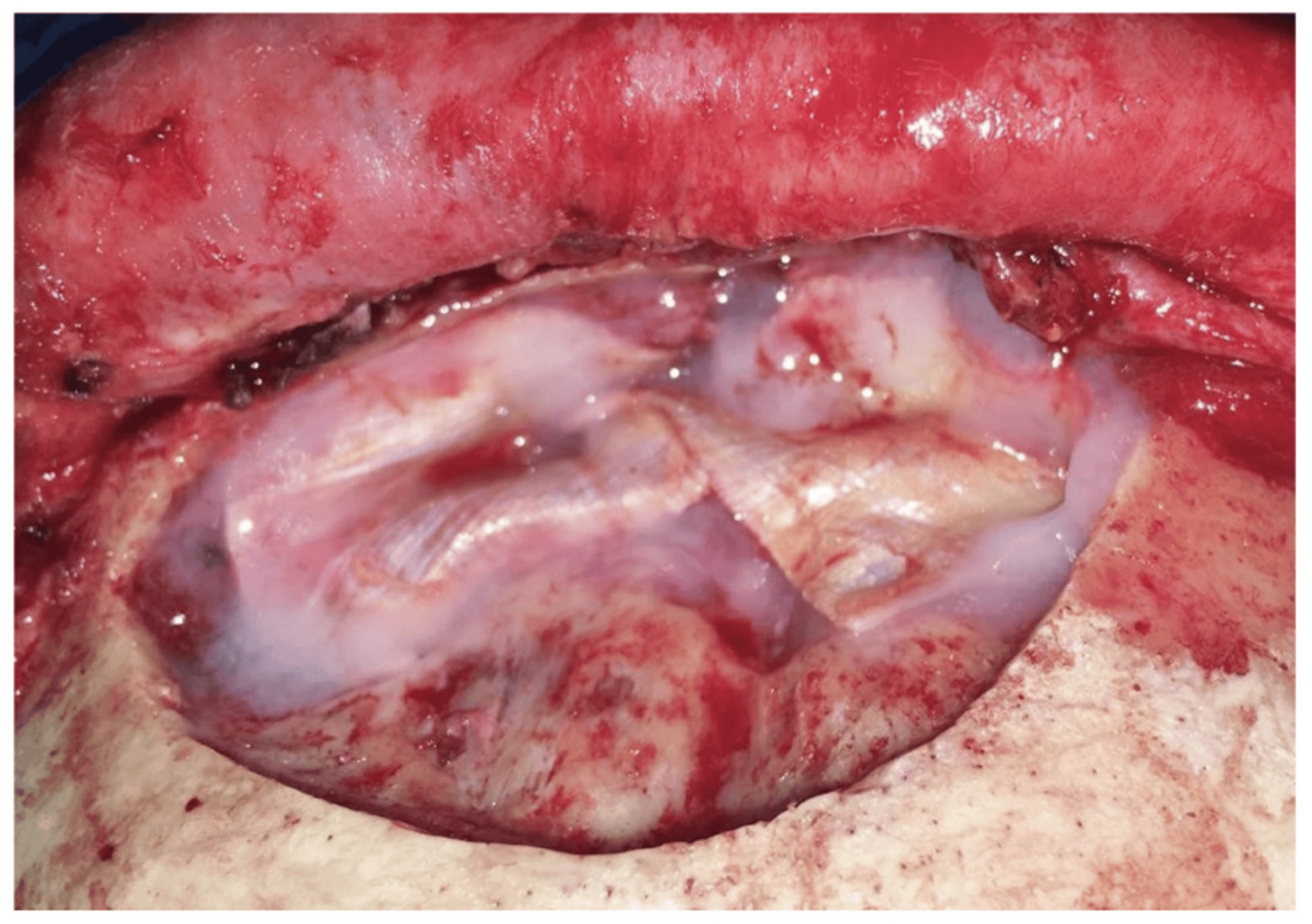
Obliteration of the frontal sinus after carefully removing the sinus mucosa and opening the ostium bilaterally.

**Figure 10:**
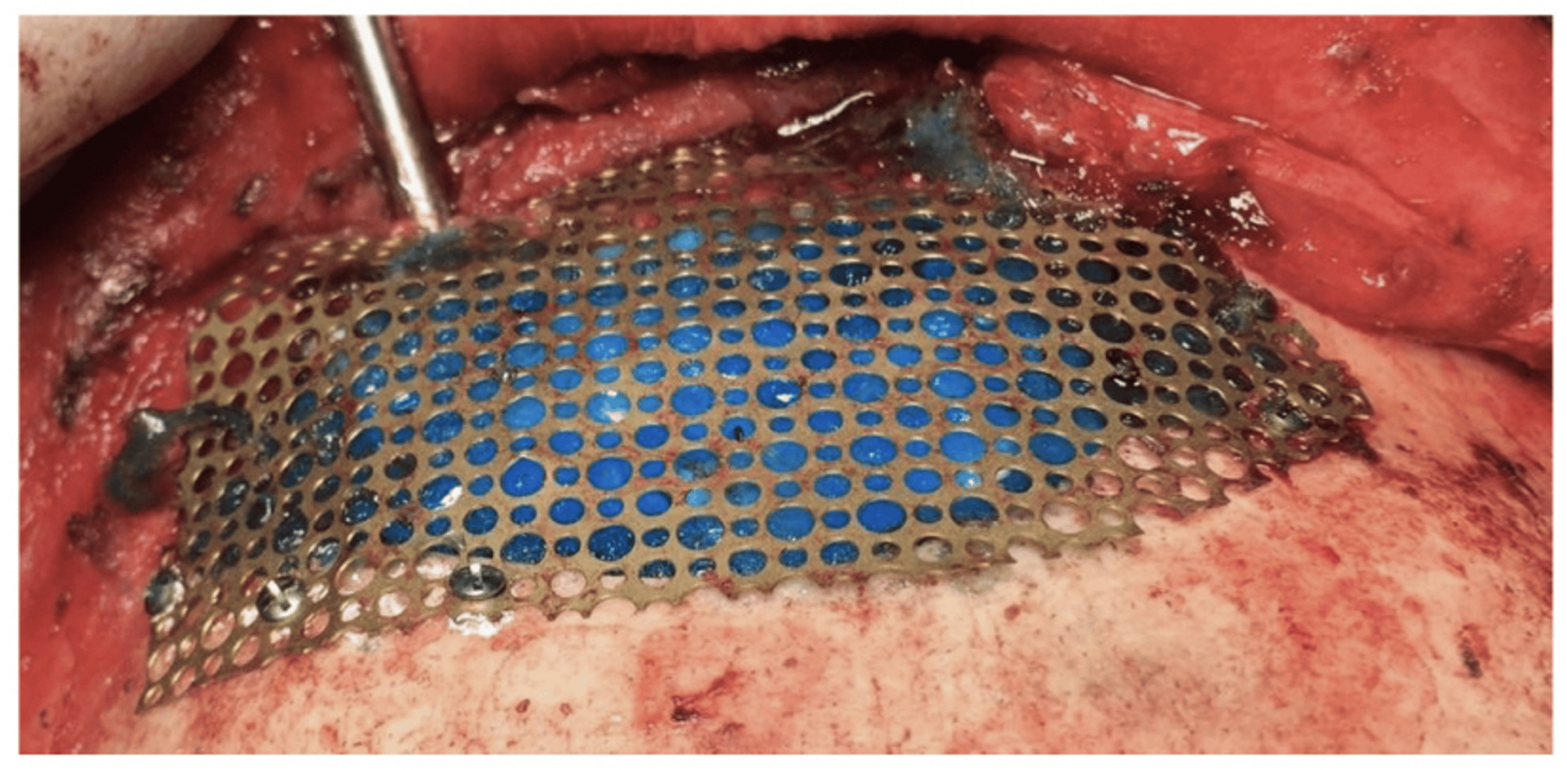
Reconstruction of the anterior wall with mesh. A dura sealant can be seen beneath the mess to prevent CSF leak due to bone erosion above the dura. CSF: cerebrospinal fluid

**Figure 11:**
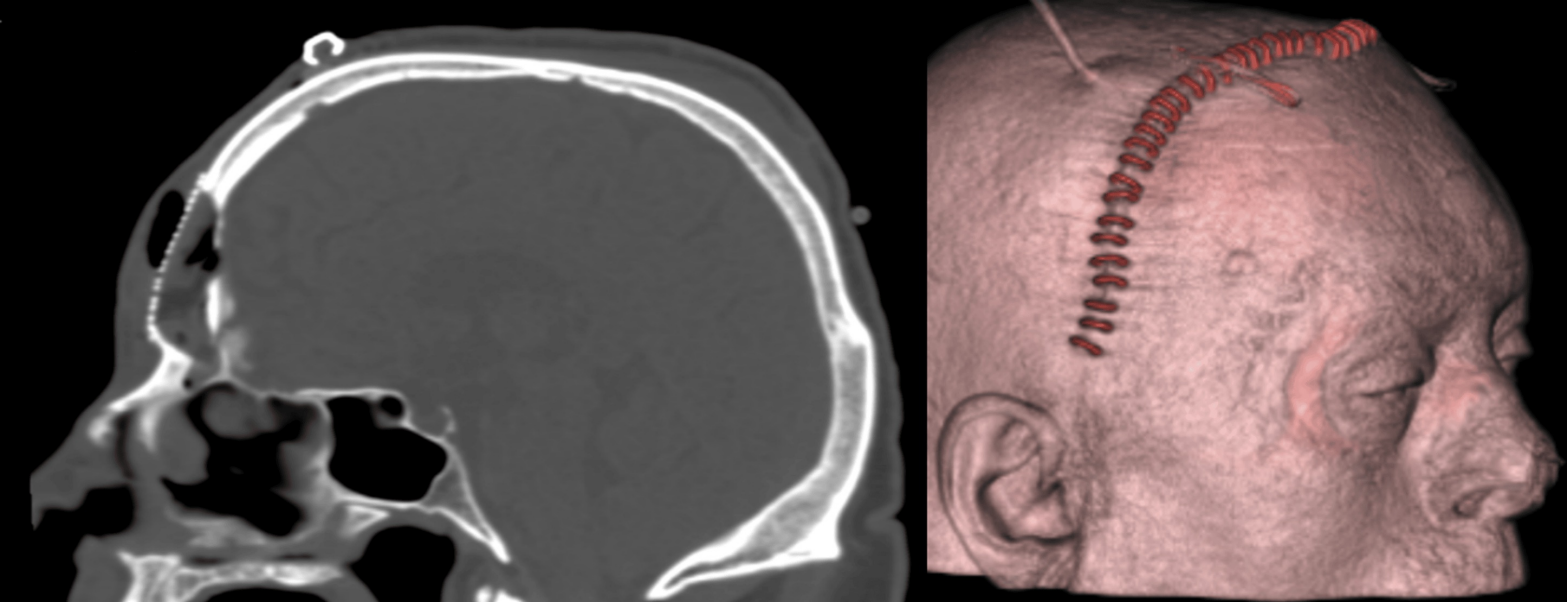
Post-op sagittal CT scan and surface rendering after the removal of the mass and bone reconstruction. CT: computed tomography

The patient was placed in a supine position with the head placed on a three-pin skull clamp (Figure 3). A bifrontal skin incision was performed with pericranium preservation and harvesting (Figures 4-5). A liquified content of the mucocele was recognized coming through the bone erosion of the anterior wall of the frontal sinus (Figures 6-7). Further drilling of the anterior wall revealed the posterior wall of an enlarged frontal sinus (Figure 8). Following that, a careful removal of the sinus mucosa (which was sent for histopathological examination) and opening of the ostium was done bilaterally. There was not a clear penetration of the dura at any point, but we could recognize the dura in two sites where the posterior wall was thin. For the abovementioned reasons, we decided to carefully proceed with an obliteration procedure to reduce cerebrospinal fluid (CSF) leak risk rather than leave the frontal recesses open. The risk of recurrence was eliminated as the whole mucosa was meticulously removed from both the sinus cavity and the frontal recesses. The frontal sinus was filled with fat graft and fascia lata followed by the pericranium (Figure 9). Finally, the reconstruction of the anterior wall was completed with titanium mesh, intraoperatively cut in order to fit the patient's frontal bone deficit, and a dura sealant was used beneath the mess to prevent CSF leak due to bone erosion above the dura (Figure 10). The patient recovered well after the surgery, was neurologically intact, and was discharged on postoperative day 2. The histology result confirmed our initial hypothesis that the lesion in our case was a frontal mucocele. At one-year follow-up, there are no recurrence of the mucocele and an excellent cosmetic result (Figure 11).

## Discussion

Paranasal mucoceles are slow-growing cystic formations filled with mucoid material. They most commonly arise from the frontal sinus, usually due to previous infection, trauma, surgery, or tumors such as osteomas [1]. These lesions are lined with the epithelium, and they are caused by the continuous or intermittent obstruction of the sinus ostia and draining duct [8].

In the case of frontal sinus mucoceles, blockage of the draining ducts results in the accumulation of mucosa secretions and the development of the cyst. This cystic lesion slowly grows towards the path of the least resistance which is usually the anterior wall of the frontal sinus and the orbital walls. Consequently, the anterior wall is thinned and finally eroded due to chronic pressure from the cyst as in our case.

A detailed clinical history, along with CT and MRI scans, can be helpful to diagnose this entity before the histopathological confirmation since other entities should be considered in the differential diagnosis such as mucus retention cyst, antrochoanal polyp, acute sinusitis, and fungal mycetoma which however do not typically cause bone expansion. Paranasal sinus tumors such as inverted papilloma or sinonasal carcinoma, although extremely rare, should not be overlooked. CT scan reveals a well-defined, homogeneous isodense lesion with bone erosion and marginal sclerosis. Surgical treatment involves the careful extirpation of the mucocele and cranialization of the sinuses and/or complete obliteration of them [3]. Upon finding the draining duct, its obliteration should be carried with attention, in order to reduce the risk of any recurrence to the minimum [9]. Meticulous reconstruction is of paramount importance upon dealing with this pathology using fat grafts, fibrin glue, and pericranium and titanium mesh plates in appropriate sizes during the transcranial approach. Other techniques have also been described in the current literature in recurrent mucoceles using the endoscopic approach and osteoplastic flaps [2]. In this case, we opted for the transcranial access for two main reasons: (1) the neurosurgical and craniofacial team were more familiar with the open technique and (2) this approach can provide the same efficiency as the other techniques when following the basic principles for obliterating the mucocele and for proper reconstruction.

## Conclusions

Frontal mucoceles are a well-known pathology that requires meticulous repair following basic reconstruction rules so that the recurrence rate is eliminated. As presented in our case report, its development can be extremely delayed following head trauma. In our opinion, surgical treatment is prompt since it can achieve both excellent obliteration and cosmetic results.
